# Gonadotropin-releasing hormone agonists, anti-androgens and the risk of cardio-cerebrovascular disease in prostate cancer patients: an asian population-based observational study

**DOI:** 10.7150/jca.38237

**Published:** 2020-04-06

**Authors:** Jong-Mi Seong, Dongho Shin, Jae Woo Sung, Shinjay Cho, Jonghyup Yang, Sungmin Kang, Hyong Woo Moon, Kyu Won Lee, U-Syn Ha

**Affiliations:** 1 Ewha womans university, Department of pharmacy, Seoul, Republic of Korea; 2 Department of Urology, Seoul St. Mary's Hospital, College of Medicine, The Catholic University of Korea, Seoul, Republic of Korea; 3 Department of Urology, Catholic Kwandong University College of Medicine, International St. Mary's Hospital, Incheon, Republic of Korea.

**Keywords:** Prostatic Neoplasms, Gonadotropin-Releasing Hormone, Antiandrogens, Cerebrovascular Disease, Cardiovascular Diseases

## Abstract

**Purpose**: To conduct a population-based study to determine whether the use of GnRH agonist and antiandrogens are associated with an increased risk of cardio-cerebrovascular disease (CCVD) in Asian patients with prostate cancer using the National Health Insurance Service-Elderly Cohort Database (NHIS-ECD).

**Materials and Methods**: We included a total of 2,413 men aged 60 years or older with prostate cancer between January 2003 and December 2008. Outcomes of interest included the first occurrence of cardiovascular events [acute myocardial infarction (AMI), ischemic heart disease (IHD)] and cerebrovascular events [ischemic stroke (IS), and cerebrovascular disease (CVD)].

**Results**: The 5-year AMI-free rates of patients diagnosed with prostate cancer and treated with GnRH agonists, antiandrogens alone, or androgen deprivation therapy (ADT)-naïve interventions were 97.0%, 96.5%, and 98.3%, respectively, while the 5-year IHD-free rates were 93.2%, 92.3%, and 94.5%, respectively. Exposure to GnRH agonists or antiandrogen regimens did not significantly increase the risk of AMI or IHD compared to ADT-naïve treatment in multivariate Cox proportional-hazards models after adjusting for other covariates. Five-year IS-free rates of patients exposed to GnRH agonists, antiandrogens alone, and those with ADT-naïve prostate cancer were 94.8%, 94.7%, and 95.5%, respectively, while the five-year CVD-free rates were 92.9%, 93.3%, and 94.6%, respectively. Cox proportional-hazards models also failed to show that men who received GnRH agonist or antiandrogen treatment alone carried a significantly increased risk for IS or CVD compared to ADT-naïve patients.

**Conclusions**: The current study based on Asian population suggests that treatment with neither GnRH agonist nor antiandrogens increases the risk of cardio-cerebrovascular disease compared to patients with ADT-naïve prostate cancer.

## Introduction

Androgen deprivation therapy (ADT) is routinely used to treat patients with advanced prostate cancer (PCa)^1^. It is well known that ADT causes side effects such as hot flushes, fatigue, decreased libido, and erectile dysfunction 2. Recent multiple observational studies have shown that men treated with ADT are at an increased risk of developing cardio-cerebro-vascular disease (CCVD), including myocardial infarction (MI) and ischemic stroke (IS) 3-6. These studies have led to issues and advisories regarding the relevance of ADT to CCVD 7. The assumed biological mechanism underlying these observational studies is the link between ADT and the formation of atherosclerotic plaque. Recent animal studies suggest that ADT contributes to the development of atherosclerosis 8, 9. ADT induces metabolic changes that promote the development and progression of atherosclerotic plaques under the effect of hormonal factors on plaque growth, rupture, and thrombosis 10.

However, these theories remain inconclusive. Further studies are needed to confirm the association between ADT and CCVD. In particular, physiological, genetic, and ethnic differences might contribute to metabolic changes associated with ADT. However, most studies investigating the association between ADT and CCVD involved the Western population. Only a few studies involved Asian populations, including a small number of patients in a single institution, which does not represent the whole population.

Therefore, this population-based study used the National Health Insurance Service-Elderly Cohort Database (NHIS-ECD). To determine whether the use of ADT was associated with an increased risk of CCVD in patients with PCa.

## Patients and Methods

### Data source

This study used claims data acquired from NHIS-ECD released by the Korean National Health Insurance Service (KNHIS). The KNHIS provides data sharing service to support policy and academic investigations using claims data collected under the national health insurance program. The cohort members of NHIS-ECD were selected by a simple random sampling method from patients aged 60 years or older who qualified for the health insurance program in 2012. These cohort members were followed up for 12 years until 2013 unless the individual was disqualified due to death or emigration. The NHIS-ECD contains information regarding 558,147 elderly patients, accounting for approximately 10% of the entire South Korean patients aged 60 years or older. The following variables were included in the NHIS-ECD data: socio-economic status (including death and disability), principal diagnosis or comorbidities based on the International Classification of Disease, 10th Revision (ICD-10), surgical procedures, and drug prescription (generic name, dosage, and prescription duration). Because patient data in NHIS-ECD were fully de-identified, the requirement for Institutional Review Board approval was waived.

### Study cohort

**Figure 1:**
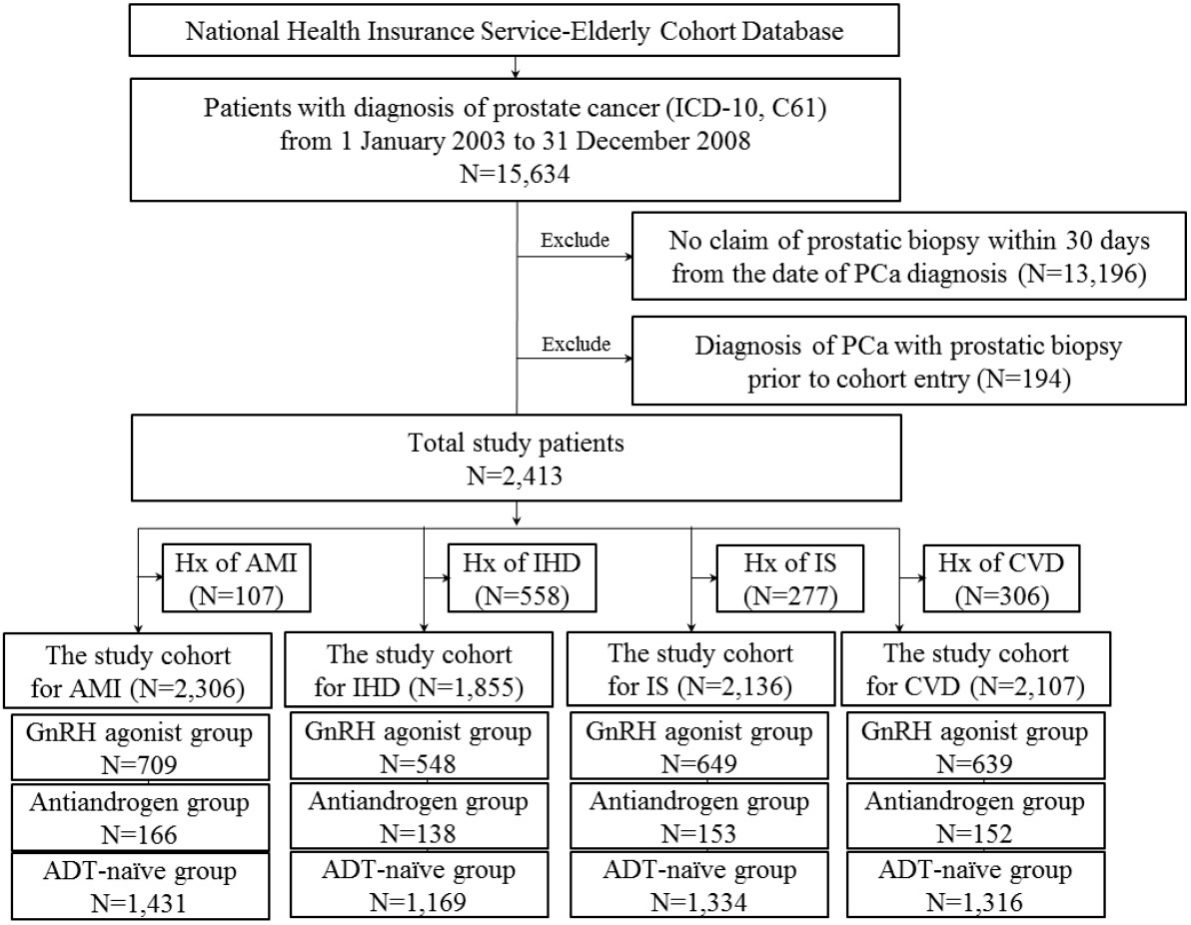
Flow diagram for selection of the study cohort.

Male patients aged 60 years or older with a first diagnosis of PCa between January 2003 and December 2008, and prostatic biopsy within 30 days from the date of the first diagnosis were included in this study (Figure 1).

Patients with prevalent CCVD who had diagnosis of either condition prior to cohort entry or 6 months after cohort entry were excluded. Study subjects were classified according to treatment type. Patients treated with gonadotropin-releasing hormone [GnRH] agonists within 6 months of PCa diagnosis were defined as GnRH agonist users. Patients treated with antiandrogens alone within six months of diagnosis were defined as antiandrogen users. We included patients who were treated for at least 6 months (GnRH agonists or antiandrogens only). Patients who received neither type of treatment were defined as ADT-naïve.

### Outcomes and Covariates

CCVD events included cardiovascular and cerebrovascular events. Cardiovascular events were classified into acute myocardial infarction (AMI) and ischemic heart disease (IHD) while cerebrovascular events were classified into ischemic stroke (IS) and cerebrovascular disease (CVD). Outcomes of interest included the first occurrence of AMI, IHD, IS, and CVD. Incident AMI was defined as a hospitalization or emergency room (ER) visit with an ICD-10 code of I21-I23 or I25.2 in the primary or secondary diagnosis code. Similarly, IHD, IS, or CVD was defined as a hospitalization or ER visit with an ICD-10 code of I20-I25 for IHD, I63 for IS, and I60-I69 for CVD in the primary or secondary diagnosis code.

Baseline covariates were derived from medical claims during 12 months preceding the cohort entry. The variables included age, year of diagnosis of PCa, diabetes mellitus (ICD-10 code of E10-14), hypertension (ICD-10 code of I10, I11, I13, and I14), and Charlson comorbidity index (CCI). CCI was calculated from medical claims one year before PCa diagnosis using the method proposed by Quan et al 11.

### Statistical analysis

Each patient was followed up from the cohort entry date until the diagnosis of the first CCVD event of interest, the start of another ADT, death, or the end of study period (31 December 2013). Analysis of variance (ANOVA) was used to compare means while Chi-square test was used to compare the proportions of study patients according to the different treatment regimens. We calculated the incidence rates of AMI, IHD, IS, and CVD during treatment with GnRH agonists, antiandrogens alone, or ADT-naïve interventions. Survival analyses were performed using Cox proportional-hazards regression. All covariates in Table 1 were adjusted to calculate the hazard ratios and their 95% confidence intervals. All analyses were performed using the SAS software version 9.4 (SAS Institute, Cary, NC, USA).

## Results

The current study cohort comprising a total of 2,413 prostate cancer patients included 738 who received GnRH agonist, 173 treated with antiandrogen alone, and 1502 exposed to neither type of treatment. The development of IHD, AMI, IS, and CVD in the study cohort was followed for a median of 5.79 years (range, 33 days to 11.0 years), 5.84 years (range, 33 days to 11.0 years), 5.62 years (range, 4 days to 11.0 years), and 5.50 years (range, 4 days to 11.0 years) following PCa diagnosis, respectively.

### Overall patient characteristics

The overall patient characteristics in the prostate cancer study cohort are summarized in Table 1. Significant baseline differences were found in age, CCI, and BMI between ADT cohort and ADT-naïve cohort. Patients in the ADT cohort (GnRH agonist or antiandrogen only) were more likely to be older with a higher CCI. They also showed a distinct difference in age distribution. More than 20% of the ADT cohort were men aged above 80 years. Conversely, only 7.7% of men in the ADT-naïve cohort were aged above 80 years.

### Analysis of cardiovascular event outcome

**Figure 2:**
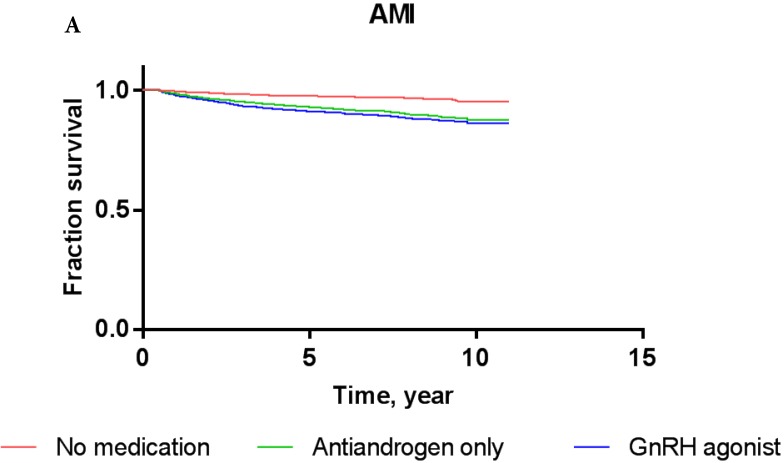

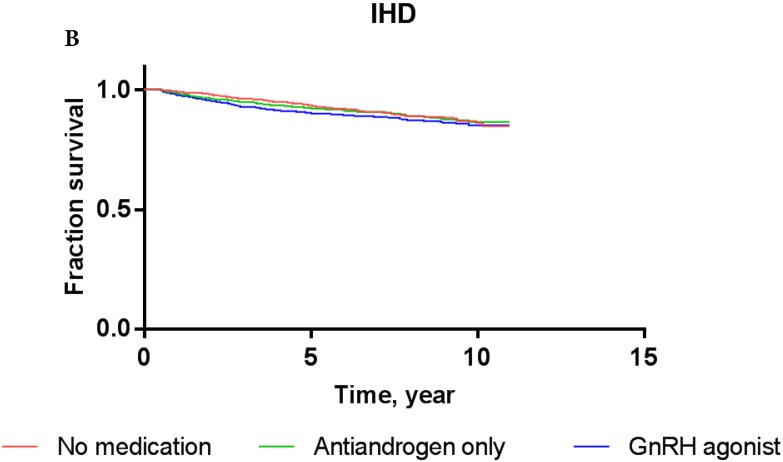
Kaplan-Meier survival curves for patients with AMI (A) and IHD (B) according to prostate cancer treatment modality.

Overall, the 5-year AMI-free and IHD-free rates were 97.8% and 94.0%, respectively, in the current cohort. The AMI-free rates of patients treated with GnRH agonists, antiandrogens, and those who were ADT-naïve were 97.0%, 96.5%, and 98.3%, respectively, while the IHD-free rates were 93.2%, 92.3%, and 94.5%, respectively (Figure 2). Results of Cox proportional-hazards analysis stratified into AMI and IHD in the current cohort are shown in Table 2.

In crude Cox proportional-hazards models, the use of GnRH agonist in patients resulted in a significantly increased risk of AMI (hazard ratio/HR: 1.996, *P* = 0.01) compared to ADT-naïve patients (Table 2) as shown by the Kaplan-Meier survival curve (2A; log-rank *P*-value = 0.04). Conversely, treatment with antiandrogen alone was not associated with a significantly increased risk of AMI (HR: 1.799; *P* = 0.27). In multivariate Cox proportional-hazards models, the use of GnRH agonists (HR: 1.503; *P* = 0.17) or antiandrogen only (HR: 1.208; P = 0.73) in PCa patients did not significantly increase the risk of AMI after adjusting for other covariates compared to ADT-naïve patients.

Concerning IHD, the Cox proportional hazards models showed that PCa patients who received GnRH agonist did not have a significantly increased risk compared to ADT-naïve patients in crude analysis (HR: 1.257; P = 0.24) or multivariate analysis (HR 1.107; P = 0.62). The use of antiandrogen alone did not show a significantly increased risk of IHD compared to ADT-naïve patients in crude analysis (HR: 1.540; P = 0.22) or multivariate analysis (HR: 1.337; P = 0.42). These results were consistent with Kaplan-Meier survival curves, showing no significant difference in the survival of patients exposed to GnRH agonist or antiandrogen alone compared to ADT-naïve group in each event category (P = 0.29, log-rank test for IHD).

### Analysis of cerebrovascular event outcome

**Figure 3:**
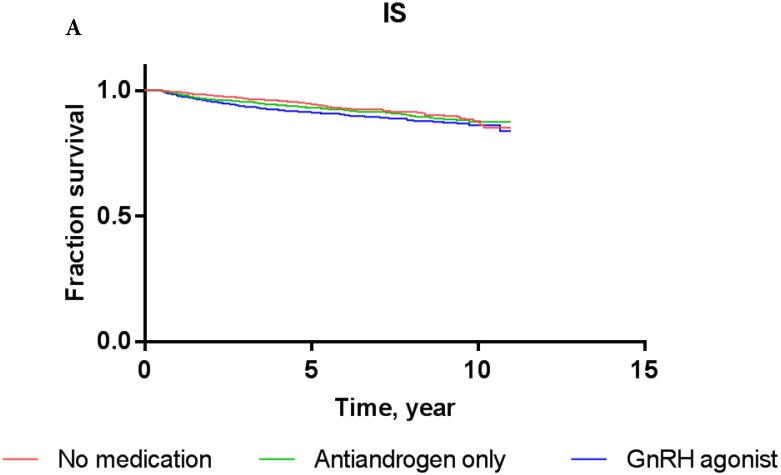

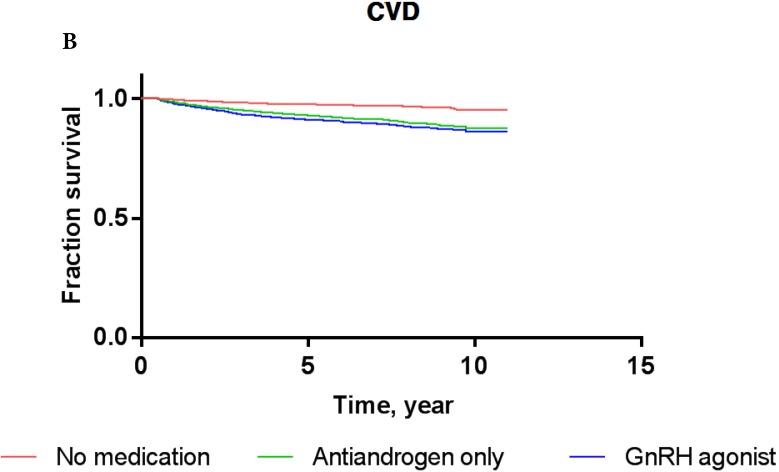
Kaplan-Meier survival curves for patients with IS (A) and CVD (B) according to prostate cancer treatment modality.

Overall, the 5-year IS-free and CVD-free rates were 95.3% and 94.1%, respectively. The IS-free rates for PCa patients exposed to GnRH agonist, antiandrogens, and those who were ADT-naïve were 94.8%, 94.7%, and 95.5%, respectively, while the CVD-free rates were 92.9%, 93.3%, and 94.6%, respectively (Figure 3).

The results of Cox proportional-hazards analysis stratified into IS and CVD in the current cohort are shown in Table 3. The Cox proportional-hazards models showed that PCa patients who were treated with GnRH agonist did not carry a significantly increased risk of IS or CVD compared to ADT-naïve patients in crude analysis (for IS: HR, 1.156; P = 0.4633; for CVD: HR, 1.111; P = 0.5579) or multivariate analysis (for IS: HR, 0.978; P = 0.9128; for CVD: HR, 0.939; P = 0.7339). PCa patients who used antiandrogens alone did not show a significantly increased risk of IS or CVD either compared to ADT-naïve patients in crude analysis (for IS: HR, 1.001; P = 0.9981; for CVD: HR, 0.926; P = 0.8435) or multivariate analysis (for IS: HR, 0.822; P = 0.6484; for CVD: HR, 0.760; P = 0.4891). These results were consistent with Kaplan-Meier survival curves (Figure 3), showing no significant difference in survival of patients exposed to GnRH agonist or antiandrogen only compared to ADT-naïve patients in each event category (for IS: log-rank test* P*-value = 0.76; for CVD: log-rank test *P*-value = 0.81).

## Discussion

The key findings of this Asian population-based study are as follows. First, prostate cancer patients who received GnRH agonist or antiandrogens only did not show an increased risk of cardiovascular disease compared to ADT-naïve patients. Second, the use of GnRH agonist or antiandrogens was not a significant risk factor for developing cerebrovascular disease in Asian men with prostate cancer.

Despite ongoing review, the FDA drug safety communication has warned that ADT increases the risk of certain cardiovascular diseases, and physicians should monitor their development 12. These recommendations are consistent with the Science Advisory issued by the American Heart Association, American Cancer Society, and American Urological Association. Recent meta-analyses using observational data of ADT and the risk of CCVD events in men with prostate cancer have also suggested that ADT might increase the risk of CCVD 13, 14. The pathogenesis of ADT-induced CCVD may involve changes in body composition and metabolism. ADT has been shown to suppress testosterone production, which leads to decreased lean muscle mass but increased fat mass 15. Another suggested mechanism is that the low level of testosterone induces insulin resistance and the adverse lipid profile, which increase vessel wall thickness and trigger atherosclerosis, resulting in endothelial dysfunction 16, 17.

However, the relationship between ADT and CCVD remains controversial. In addition, most studies investigating the effect of ADT on CCVD have been based on western populations including Caucasians. As mentioned above, the pathophysiological events underlying changes in body composition, especially fat mass, play a key role in the development of CCVD. Significant differences in body composition and fat mass have been found between western population and Asians. In general, the prevalence of obesity is significantly higher in western population than in Asian population 18. In the United States, 33% of the population is obese. In the UK, 26.9% of the residents are obese. In Korea and Japan, the percentage of obese people is relatively very low 19. A small amount of fat mass in the Asian population has an impact on the risk of CCVD following ADT.

Physiological and genetic differences according to ethnicity also affect the relationship between ADT and CCVD. Differences in androgen receptor (AR) CAG repeat length polymorphism 20-22 and deletion of UGT2B genes 23, 24 contribute to differences in the development of CCVD between Asians and Caucasians. These ethnic differences affect the development of CCVD following ADT. Therefore, further investigations are needed to determine the association between ADT and CCVD in Asian population.

Our results are in agreement with previous Asian population-based studies, including the Japanese population 25 and the Taiwanese population 26. The Japanese population study showed no increased risk of coronary heart disease in patients exposed to ADT. The Taiwanese population study showed no increased risk of IS following ADT 27. However, other Asian population studies suggested increased risks of CCVD in patients receiving ADT 28-30. Although such discrepancies cannot be explained, studies reporting a positive association between ADT and CCVD involve relatively small numbers of subjects at a single institution. Therefore, they cannot accurately represent the population. The current study is a nationwide representative analysis of Korean population. This study analyzed data from NHIS-ECD, which sampled more than 55 million people aged 60 years or older through national health insurance service registration. The study represents approximately 10% of all South Korean patients aged 60 years or older. It therefore provides a highly representative cohort of the Korean population.

It is worth noting that this is the first population-based observational study to investigate the relationship between ADT and the risk of CCVD among Koreans, a population with homogeneous ethnicity. To the best of our knowledge, the sample size and observation period in this study are larger than those of previous studies involving Asian population. In addition, we carefully defined the potential confounders at baseline. Male patients with a history of any cardiovascular disease (except hypertension, ischemic, or hemorrhagic stroke) and renal failure were excluded from the analysis, which was thus more reliable by controlling potential confounders. In addition, those who were aged above 40 years in South Korea were advised to undergo health examination including electrocardiography and lipid profile. Any abnormal findings were covered by NHIS for further medical evaluation. Therefore, the analysis entailing frequent and widespread medical evaluation has considerable strength and power.

Our study has some limitations due to potential confounding factors associated with lifestyle. Smoking status and obesity are known risk factors for CCVD. However, they were not evaluated in the current study. Another limitation was that patients were not categorized by cancer status such as stage, metastasis, and Gleason score in the current study because cancer profile was unavailable in the current database. However, the extent or staging of prostate cancer might not modify CCVD risk. Lastly, the potential for disease misclassification based on claim database was another limitation. The Korean NHISS covers almost all medical costs of patients with major diseases such as malignancy, coronary heart disease, and stroke. Patients only need to pay 5% of the total medical cost. However, the registration and certification criteria for patients with major diseases are stringent, which ensures sufficient reliability and trustworthiness of medical claims.

## Conclusions

The current study based on nation-wide population in Korea suggests that the use of ADT in prostate cancer patients does not increase the risk of CCVD compared to ADT-naïve patients. Although it is not sufficient to establish the association between ADT and CCVD, the Asian population differs from western population. Therefore, further well-designed studies including different regions and populations are needed to elucidate the increased risk of CCVD following ADT.

## Figures and Tables

**Table 1:** Overall patient characteristics of the cohorts

		ADT-naïve(N=1502)		GnRH agonists(N=738)		Antiandrogen only(N=173)		p-value
		N	%	N	%	N	%	
Age	Mean, SE	71.1	5.5	74.2	6.1	74.1	6.7	< 0.01
Age group	60-69	655	43.6	190	25.7	49	28.3	
	70-79	731	48.7	393	53.3	83	48.0	
	80 <	116	7.7	155	21.0	41	23.7	
Year of diagnosis of prostate cancer	2003	205	13.6	71	9.6	35	20.2	< 0.01
	2004	245	16.3	89	12.1	28	16.2	
	2005	208	13.8	108	14.6	24	13.9	
	2006	247	16.4	119	16.1	32	18.5	
	2007	280	18.6	162	22.0	25	14.5	
	2008	317	21.1	189	25.6	29	16.8	
Comorbidity	Diabetes	432	28.8	227	30.8	51	29.5	0.62
	Hypertension	817	54.4	430	58.3	90	52.0	0.14
CCI	Mean, SE	4.2	2.3	4.9	3.0	4.8	2.9	< 0.01
	2	339	22.6	144	19.5	38	22.0	
	3	339	22.6	155	21.0	35	20.2	
	4	294	19.6	135	18.3	36	20.8	
	5<	470	31.3	304	41.2	64	37.0	
Comorbidities in CCI	Myocardial infarction	40	2.7	18	2.4	6	3.5	0.75
	Congestive heart failure	75	5.0	57	7.7	15	8.7	0.01
	Peripheral vascular disease	145	9.7	102	13.8	11	6.4	< 0.01
	Cerebrovascular disease	228	15.2	142	19.2	28	16.2	0.05
	Dementia	20	1.3	18	2.4	3	1.7	0.16
	Chronic pulmonary disease	557	37.1	254	34.4	72	41.6	0.17
	Rheumatic disease	74	4.9	38	5.1	6	3.5	0.65
	Peptic ulcer disease	523	34.8	273	37.0	44	25.4	0.02
	Mild liver disease	331	22.0	177	24.0	36	20.8	0.5
	Diabetes without chronic complication	404	26.9	208	28.2	47	27.2	0.81
	Diabetes with chronic complication	136	9.1	76	10.3	13	7.5	0.44
	Hemiplegia or paraplegia	22	1.5	19	2.6	4	2.3	0.17
	Renal disease	35	2.3	23	3.1	5	2.9	0.53
	Moderate or severe liver disease	13	0.9	7	0.9	2	1.2	0.92
	Metastatic solid tumor	81	5.4	107	14.5	29	16.8	< 0.01
	AIDS/HIV	0	0.0	0	0.0	0	0.0	-

**Table 2:** Crude- and multivariable-adjusted hazard ratios for cardiovascular disease according to treatment modality in prostate cancer cohort

	Treatment group	N	Event	Person year	Incidence	H.R (95% Confidence interval)			
						Crude		Adjusted*	
**IHD**	**No medication**	1169	84	7128.83	11.78	1	Ref.		Ref.
	**GnRH agonists**	548	39	2723.23	14.32	1.257	(0.858-1.840)	1.107	(0.743-1.651)
	**Antiandrogen only**	138	9	536.26	16.78	1.54	(0.774-3.067)	1.337	(0.661-2.706)
**AMI**	**No medication**	1431	29	8865.31	3.27	1	Ref.		Ref.
	**GnRH agonists**	709	23	3549.95	6.48	1.996	(1.151-3.461)	1.503	(0.841-2.688)
	**Antiandrogen only**	166	4	681.74	5.87	1.799	(0.631-5.127)	1.208	(0.413-3.529)

* Adjusted for all covariated in Table 1

**Table 3:** Crude- and multivariable-adjusted hazard ratios for cerebrovascular disease according to treatment modality in prostate cancer cohort

	Treatment group	N	Event	Person year	Incidence	H.R (95% Confidence interval)			
						Crude		Adjusted*	
IS	No medication	1334	86	8185.07	10.51	1	Ref.		Ref.
	GnRH agonists	649	37	3200.95	11.56	1.156	(0.785-1.702)	0.978	(0.655-1.460)
	Antiandrogen only	153	6	609.68	9.84	1.001	(0.437-2.292)	0.822	(0.354-1.910)
CVD	No medication	1316	107	8039.8	13.31	1	Ref.		Ref.
	GnRH agonists	639	44	3132.05	14.05	1.111	(0.781-1.581)	0.939	(0.652-1.352)
	Antiandrogen only	152	7	609.07	11.49	0.926	(0.431-1.990)	0.76	(0.349-1.655)

* Adjusted for all covariated in Table 1

## Abbreviations

ADT: Androgen deprivation therapy
PCa: prostate cancer
CCVD: cardio-cerebro vascular disease
MI: myocardial infarction
IS: ischemic stroke
NHIS-ECD: National Health Insurance Service-Elderly Cohort Database
KNHIS: Korean National Health Insurance Service
ICD-10: the International Classification of Disease, 10th Revision
GnRH: gonadotropin releasing hormone
AMI: acute myocardial infarction
IHD: ischemic heart disease
IS: ischemic stroke
CVD: cerebrovascular disease
ER: emergency room
CCI: Charlson comorbidity index
ANOVA: Analysis of variance
HR: hazard ratio
